# An e-learning application on electrochemotherapy

**DOI:** 10.1186/1475-925X-8-26

**Published:** 2009-10-20

**Authors:** Selma Corovic, Janez Bester, Damijan Miklavcic

**Affiliations:** 1 University of Ljubljana, Faculty of Electrical Engineering, Trzaska 25, 1000 Ljubljana, Slovenia

## Abstract

**Background:**

Electrochemotherapy is an effective approach in local tumour treatment employing locally applied high-voltage electric pulses in combination with chemotherapeutic drugs. In planning and performing electrochemotherapy a multidisciplinary expertise is required and collaboration, knowledge and experience exchange among the experts from different scientific fields such as medicine, biology and biomedical engineering is needed. The objective of this study was to develop an e-learning application in order to provide the educational content on electrochemotherapy and its underlying principles and to support collaboration, knowledge and experience exchange among the experts involved in the research and clinics.

**Methods:**

The educational content on electrochemotherapy and cell and tissue electroporation was based on previously published studies from molecular dynamics, lipid bilayers, single cell level and simplified tissue models to complex biological tissues and research and clinical results of electrochemotherapy treatment. We used computer graphics such as model-based visualization (i.e. 3D numerical modelling using finite element method) and 3D computer animations and graphical illustrations to facilitate the representation of complex biological and physical aspects in electrochemotherapy. The e-learning application is integrated into an interactive e-learning environment developed at our institution, enabling collaboration and knowledge exchange among the users. We evaluated the designed e-learning application at the International Scientific workshop and postgraduate course (Electroporation Based Technologies and Treatments). The evaluation was carried out by testing the pedagogical efficiency of the presented educational content and by performing the usability study of the application.

**Results:**

The e-learning content presents three different levels of knowledge on cell and tissue electroporation. In the first part of the e-learning application we explain basic principles of electroporation process. The second part provides educational content about importance of modelling and visualization of local electric field in electroporation-based treatments. In the third part we developed an interactive module for visualization of local electric field distribution in 3D tissue models of cutaneous tumors for different parameters such as voltage applied, distance between electrodes, electrode dimension and shape, tissue geometry and electric conductivity. The pedagogical efficiency assessment showed that the participants improved their level of knowledge. The results of usability evaluation revealed that participants found the application simple to learn, use and navigate. The participants also found the information provided by the application easy to understand.

**Conclusion:**

The e-learning application we present in this article provides educational material on electrochemotherapy and its underlying principles such as cell and tissue electroporation. The e-learning application is developed to provide an interactive educational content in order to simulate the "hands-on" learning approach about the parameters being important for successful therapy. The e-learning application together with the interactive e-learning environment is available to the users to provide collaborative and flexible learning in order to facilitate knowledge exchange among the experts from different scientific fields that are involved in electrochemotherapy. The modular structure of the application allows for upgrade with new educational content collected from the clinics and research, and can be easily adapted to serve as a collaborative e-learning tool also in other electroporation-based treatments such as gene electrotransfer, gene vaccination, irreversible tissue ablation and transdermal gene and drug delivery. The presented e-learning application provides an easy and rapid approach for information, knowledge and experience exchange among the experts from different scientific fields, which can facilitate development and optimisation of electroporation-based treatments.

## Background

Electrochemotherapy is an effective approach in tumor treatment employing locally applied high-voltage electric pulses in combination with chemotherapeutic drugs which enter tumor cells after their membrane has been electroporated [1,2]. Electroporation is a phenomenon of cell membrane permeability increase due to local delivery of short and sufficiently intense voltage pulses via appropriate electrodes to the target cells and tissues [3,4]. In addition to electrochemotherapy, other medical applications of electroporation are emerging at an increasing rate, such as gene electrotransfection [5,6], cell fusion [7] and irreversible tissue ablation [8] and transdermal gene and drug delivery [9]. The effectiveness of cell and tissue electroporation, and thus the effectiveness of electroporation-based therapies, depends on one hand on the parameters of the applied pulses such as amplitude, duration, number and repetition frequency and type of electrodes used and on the other hand on the characteristics of the cell and tissues to be electroporated. Depending on the electric pulse parameters used, electroporation can be reversible or irreversible. Namely, when the electric pulses are applied, local electric field (*E*) is established within the treated tissue. In order to cause structural changes in cell membrane magnitude of local electric field need to achieve the critical reversible threshold value (*Erev*). The phenomenon is reversible until the magnitude of local electric field reaches the irreversible threshold value *Eirrev*, which causes permanent damages of the cell membrane. The reversible electroporation regime has to be assured in all applications in which the viability of cells has to be preserved, such as electrochemotherapy and particularly gene therapy [4]. On the other hand, in some medical and biotechnological applications such as irreversible tumour tissue ablation, liquid food sterilization or water treatment, the irreversible electroporation is used as a nonthermal method for efficient cell killing [10]. The key role in electroporation effectiveness plays the local electric field, which can be directly modified by the amplitude of delivered electric pulses and electrodes used for electric pulse delivery [11]. Thus, for controlled use of the method in each particular electroporation-based application electric pulse parameters and electrodes' shape and placement with respect to the target tissue need to be specifically optimized [12].

### Knowledge exchange and collaboration among the experts involved in electroporation-based therapies

In development of electroporation-based therapies (e.g. electrochemotherapy), a multidisciplinary expertise is required. In electrochemotherapy a close collaboration, knowledge and experience exchange among experts in the fields of oncology, biology, biophysics, physical chemistry and electrical, biomedical engineering and informatics is needed (Table 1). The efficacy of electrochemotherapy can be assured with the knowledge of parameters of the local electric field (i.e. pulse parameters and electrode geometry and their positioning), being crucial for successful tissue electroporation and subsequently for the best electrochemotherapy treatment outcome. Realistic mathematical models validated by corresponding experimental observations are valuable tool in designing and optimization of local electric field distribution. To develop a good mathematical model allowing for therapy outcome prediction the engineers need to posses knowledge about biological mechanisms involved in electrochemotherapy. To make the therapy as efficient as possible it is of great importance to transfer the knowledge from basic science to the field of biomedical engineering and to the practicing clinicians who performs the treatment.

**Table 1:** Scientific fields and the corresponding expertise needed in electrochemotherapy

**Field**	**Expertise**
- Oncology:	Tumor cells and tissues, cancer

- Biology:	Cells, normal tissue

- Biophysics:	Physics of biological cells and tissues

- Physical chemistry:	Chemistry

Electrical engineering:	Devices, electrodes

- Biophysical engineering:	Application of physics in medicine and biology

- Computer engineering:	Database systems, interactive web applications

Information and communication technology is necessary for efficient interdisciplinary collaboration and knowledge exchange. Internet technology has already been successfully used to support clinical trials of electrochemotherapy by establishing a central database and the Web application system for electronic collection of data (such as treatment parameters used and treatment efficiency follow up) submitted by users from distant medical centres across Europe [13-15]. Based on a comprehensive analysis of collected data, performed by the developed system the standard operating procedures for clinical electrochemotherapy of cutaneous and subcutaneous tumor in patients have been defined [2,16-18]. The clinical trials showed and numerous other studies demonstrated, that electrochemotherapy is an efficient antitumor treatment regardless of tumor histology and its location. In order to further improve the treatment planning methods also for other electroporation-based therapies, to develop the needed equipment (i.e. generators, electrodes, software) and to broaden the clinical electrochemotherapy to other types of tumours, numerous international and multidisciplinary scientific projects are being conducted.

### A collaborative e-learning in electrochemotherapy

The objective of our study was to develop an e-learning application to support collaboration, knowledge and experience exchange among experts involved in electrochemotherapy and to also apply the acquired knowledge to other electroporation-based technologies such as gene electrotransfection, irreversible tissue ablation and transdermal gene and drug delivery. The target users of our application are biomedical engineers, biologists involved in research and other application development, the clinicians, oncologists and medical personnel involved in choosing and performing the treatment, but also patients and all those who want to learn about electrochemotherapy. The target audience is therefore mixed [19] (i.e. coming from scientific areas, different fields of expertise, and with different level of experiences) and dispersed [20] (i.e. geographically located in different research centres spread around Europe/World). In order to consider the users involved in electrochemotherapy our e-learning application was designed to provide educational material for collaborative and flexible learning.

Computer-supported learning of various types i.e. e-learning based educational trainings such as web-based learning, CD-contents or virtual instruments play an important roll in sharing learning content and educational materials, which brings new potential for interdisciplinary and international co-operation among experts from different fields [21]. The e-learning programs that incorporate computer based simulations and visualization tools enable educationally effective and enjoyable learning and teaching methods compared to the conventional learning methods such as learning through listening to spoken words [22,23]. The use of computer based simulation techniques are particularly important in developing active e-learning environments and "hands-on" e-learning activities, which is proven to be important component in electromagnetic engineering, biomedical engineering and medical education [24-26]. In designing the e-learning content when the target users are coming from different professional backgrounds and with different levels of knowledge it is essential to develop an adaptive interface which can be suitable for different categories of users: novices, intermediates or expert users. In order to more clearly represent the underling mechanisms from the engineering, biological, chemical and medical sciences, scientific and information visualization concepts based on computer graphics software are necessary [27,28]. Furthermore, collaboration, learning, networking, communication of scientific ideas and knowledge and experience exchange, among the mixed and dispersed audience can be facilitated by computer-supported collaborative visualization [29,30].

The web based technologies facilitate flexible learning by providing a choice of learning modalities (i.e. in local, near or remote conditions), which is particularly important when the dispersed audience is concerned [20]. Accordingly, we used web-based technologies to collect, organize and transfer the acquired knowledge among the target audience in electrochemotherapy. We used computer graphics such as model-based visualization and simple 2D and 3D computer animations and graphical illustrations to facilitate the representation of complex biological and physical mechanisms involved in electrochemotherapy. The educational content is based on previously published results from molecular dynamics, lipid bilayers, single cell level and simplified tissue models to complex biological tissues [3,4,11,31-43].

The e-learning application is integrated into an interactive e-learning environment E-CHO [44] developed at our institution. The e-learning application on electrochemotherapy was introduced to the participants at the International Scientific workshop and postgraduate course (Electroporation Based Technologies and Treatments) that took place at the University of Ljubljana in November 2007 [45]. The pedagogical efficiency of the application was analyzed by participant evaluation on the presented educational content at the beginning and at the end of the e-learning training session. We also present the results of a simple usability evaluation of the application we performed by asking the participants to answer to a usability questionnaire and to provide users opinion/comments on the application and suggestions on its possible improvement.

## Methods

The e-learning web application is based on HTML, JavaScript, ASP and Macromedia Flash web technologies. Graphical illustrations and 3-dimensional visualizations of the electroporation process on the levels of cell membrane, cell and tissues were done by using a software package 3D Studio Max. Based on the numerical calculations of electric filed distribution carried out with software packages FEMLAB and Matlab, more simple 2-dimensional and 3-dimensional illustrations were designed using software packages 3D StudioMax, Macromedia Flash, PhotoShop and CorelDraw. The educational content (textual and graphical information) is published using Hypertext Markup Language (HTML). The designed e-learning application is integrated into E-CHO e-learning system developed by the Laboratory of telecommunications [44] (University of Ljubljana) at the Faculty of Electrical Engineering. The E-CHO e-learning environment enables the use of various types of communications among users, such as forums, e-mail correspondence and videoconferencing as well as authentication of users, statistical analysis, network traffic measurement, and support for video streaming [46].

### Evaluation

We introduced the designed e-learning application at the International Scientific workshop and postgraduate course (Electroporation Based Technologies and Treatments) [45] in order to evaluate its pedagogical and usability efficiency. The participants were a mixed audience of 17 participants with heterogeneous knowledge and experience in the field of electrochemotherapy and other electroporation-based technologies. The mixed audience was composed of participants coming from different research institutions across Europe and World:

- Denmark (University of Copenhagen: 1 biologist (PhD student) and 1 medical physician (PhD researcher) from Herlev Hospital and 1 from Gentofte Hospital);

- France (1 physicist (PhD student) from doctoral school École normale supérieure de Cachan; 1 biologist (PostDoc researcher) from Institut Gustave Roussy, Villejuif; 2 biologists (1 PhD student and 1 PostDoc researcher) from IPBS (Institut de Pharmacologie et de Biologie Structurale) - Research Unit of CNRS/UMR 5089 and University Paul Sabatier, Toulouse);

- Egypt (University of Cairo: 1 physicist (PhD student) from Biophysics Department, Faculty of Science); and

- Slovenia (University of Ljubljana: 8 electrical engineers (PhD students) from Faculty of Electrical Engineering and 1 biologist (PhD student) from Faculty of Pharmacy).

In order to statistically analyze the obtained results we divided the mixed audience/participants into two groups:

first group of 11 engineers (by gathering electrical engineers and physicists) and second group of 7 biologists (by gathering biologists and the medical physician).

The participants were gathered in a computer-based classroom providing each participant with a computer. Each of the participants was provided with a username and password to log on to the E-CHO system. Before the start of the e-learning session a Power Point presentation was presented to the participants by the instructor giving instructions on the course of studying the educational content and on the evaluation testing. In order to create a collaborative e-learning environment the participants were encouraged to collaborate (i.e. discuss between each other and with the instructor) while studying the educational content.

The participants were given the instruction to execute the e-learning session according to the linear sequence of studying steps [30] by starting at the beginning of the e-learning content and by concluding with the final evaluation tests. The evaluation tests were taken by each of the participants only once.

**Figure 1:**
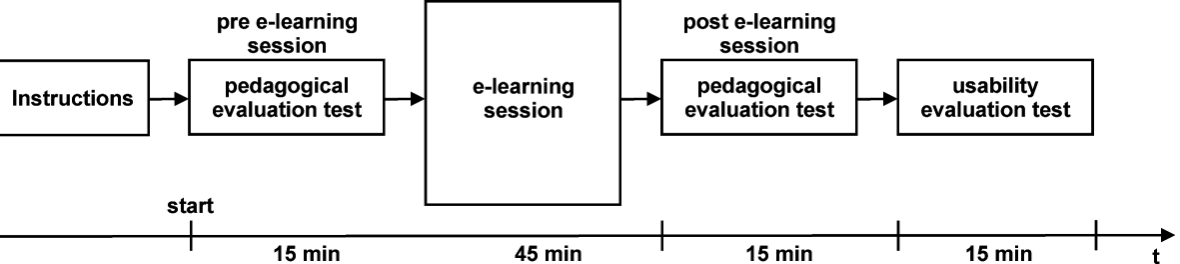
**A flow chart representing the time sequence of the steps performed during the study**.

We evaluated the e-learning application by testing the pedagogical efficiency of the presented educational content and by performing the usability study of the application. All the participants were asked to provide their agreement on the use of the results of pedagogical efficiency and usability study for the research purposes. Each of the participants individually completed the evaluation tests and submitted them to the E-CHO system for further statistical analysis. The time sequence of the steps performed during our study is given by a flow chart in Fig. 1.

### 1) Pedagogical efficiency study

In order to evaluate the pedagogical efficiency of the educational content on electrochemotherapy the participants were asked to answer to the same test before and at the end of the e-learning session. The questions were targeted so as to give 50% to 100% success. The exact questions asked in the pre and post e-learning session test are given in Additional file 1.

### 2) Usability study

The usability evaluation was conducted at the end of the e-learning session after the pedagogical efficiency evaluation was completed. The participants were asked to complete a usability questionnaire related to the user satisfaction with the developed e-learning application, in order to allow the authors (i.e. developers and instructors) to detect possible errors or to obtain the users feedback on further upgrades/improvements. The questionnaire consisted of thirteen usability related questions (see Additional file 2). The participants were asked to express their opinion on a seven point Likert scale (LS) ranging from 1 (disagree - LS (1)) to 7 (strongly agree (LS - (7)) statement or to remain neutral by checking neither agree nor disagree (NA) statement, which we considered as negative evaluation result (Additional file 2). After completing the usability questionnaire the participants were encouraged to provide their opinion/comments on the application and suggestions for its improvement.

## Results

### The structure of the e-learning content

**Figure 2:**
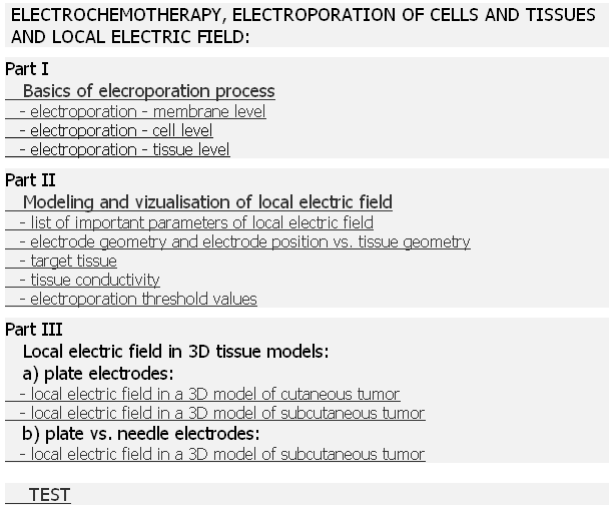
**The structure of the e-learning application on electrochemotherapy**.

The e-learning content presents three different levels of knowledge on electroporation-based treatment (i.e. electrochemotherapy) and cell and tissue electroporation. The e-learning content particularly emphasizes the importance of local electric field for successful cell and tissue electroporation. The main structure of the e-learning content is given in Fig. 2.

**Figure 3:**
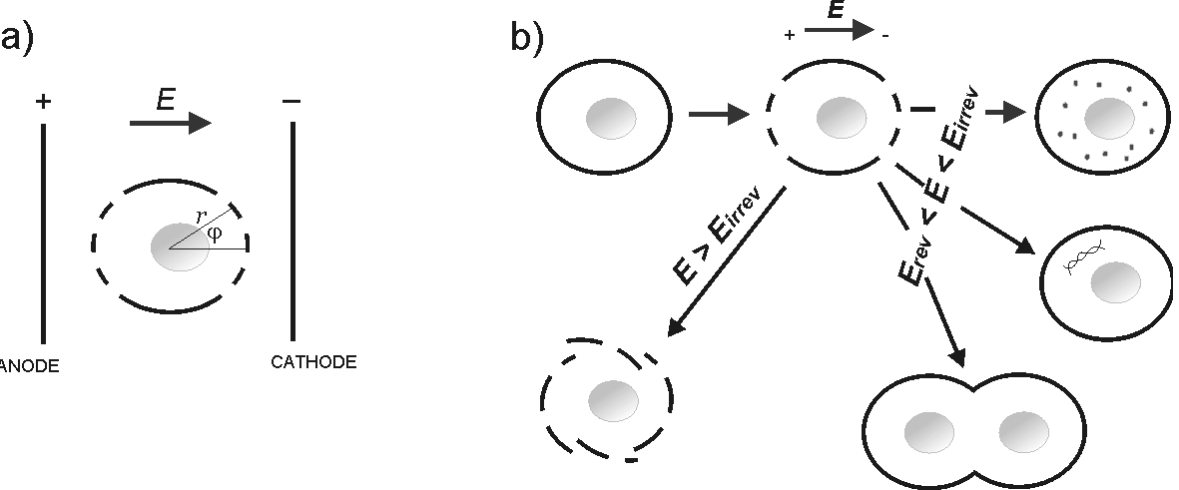
**Single cell electroporation and different electroporation regimes**. (a) The electroporation of cell membrane first occurs within the cell area facing the electrodes and (b) Different electroporation regimes: reversible *Erev *<*E *<*Eirrev *and irreversible *E *> *Eirrev*. (Redrawn from [10]).

The first part of our e-learning application (Basics of electroporation process) brings the educational material on basic mechanisms underlying electroporation process on the levels of: cell membrane, cell and tissue as a composite of cells. Electroporated cell in a local electric field exceeding reversible threshold value *E *>*Erev *is represented by a simple graphical illustration in Fig. 3a. The electroporation of cell membrane first occurs within the cell area facing the electrodes (dashed line in Fig. 3a), since the induced transmembrane potential is maximal at the poles of the cell in accordance Schwan's equation: *U*_*TI *_= -1.5 *r E *cos (*φ*), where r is the radius of the cell, E is the strength of applied electric field, and φ is the angle between the direction of the electric field and the selected point on the cell surface. Possible applications of electroporation process, depending on parameters of the electric pulses applied, are illustrated in Fig. 3b: the introduction of small molecules, macromolecules and cells' electrofusion require reversible electroporation regime (*Erev *<*E *<*Eirrev*), while the permanent cell damaging requires irreversible electroporation thus local electric field exceeding irreversible threshold *E *>*Eirrev*.

**Figure 4:**
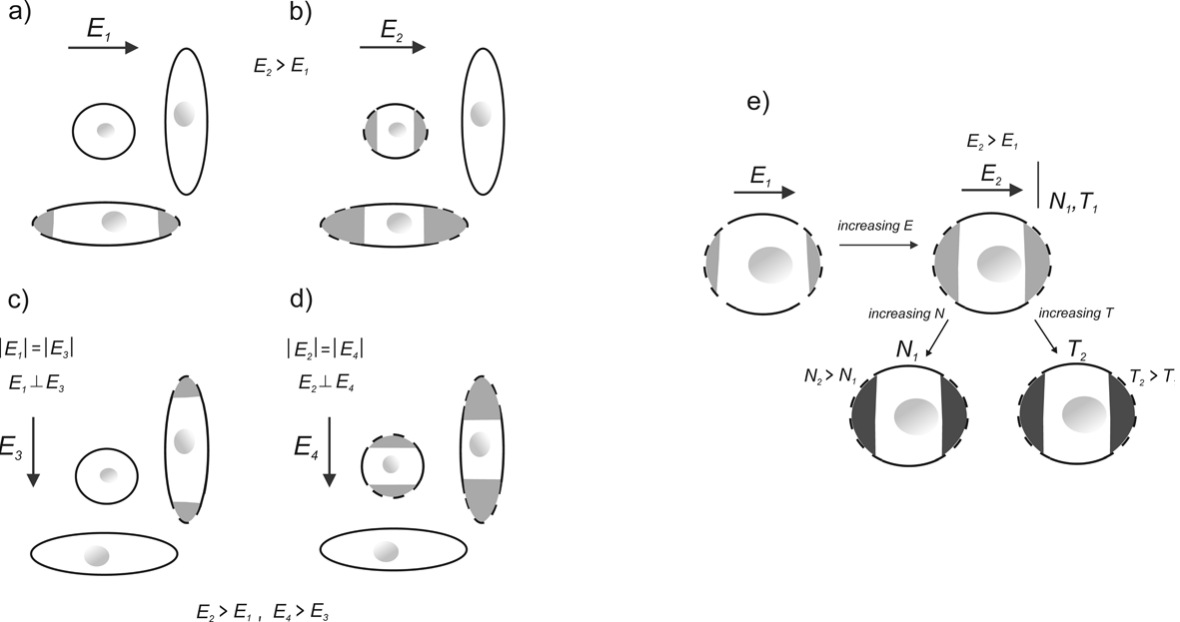
**Influence of different parameters on cell electroporation**. (a) Electric field parallel to elongated cell, (b) electric pulse amplitude is increased, (c) orientation of electric field is changed, (d) electric pulse amplitude is increased and (e) increasing the pulse amplitude results in larger area of membrane with smaller extent of electroporation, while increase in pulse number or duration does not affect the membrane area but increases the extent of electroporation. (Redrawn from [10]).

The value of induced transmembrane voltage and thus the cell electroporation depends on the cell size, shape, and the position of the cell with respect to the direction of applied electric field, which we represented in Figs. 4a, b, c and 4d. For a spheroidal cell, maximum induced transmembrane potential strongly depends on its orientation with the respect to the electric field. It is the highest when the spheroidal cell is parallel to the applied electric field. In Fig. 4e we illustrated that increasing the pulse amplitude results in larger area of membrane with smaller extent of electroporation, while increase in pulse number or duration does not affect the membrane area but increases the extent of electroporation.

**Figure 5:**
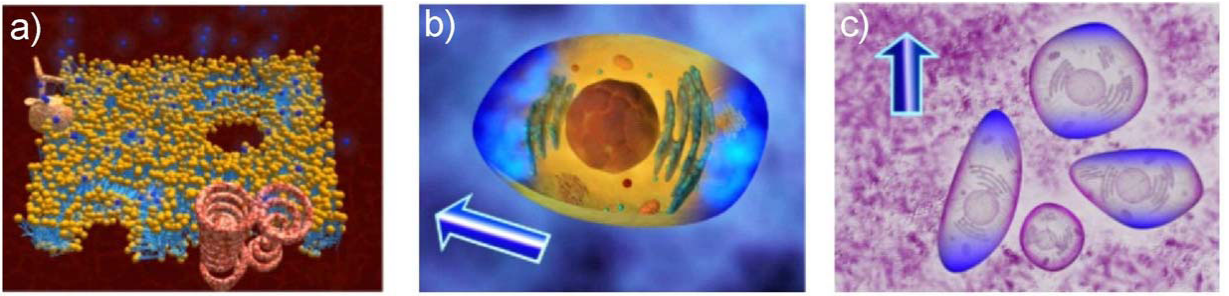
**Administration of small molecules by electroporation**. Administration of small molecules (blue molecules) through an electroporation cell membrane (a) into an electroporated cell (b) and into the successfully electroporated cells within an exposed tissue (i.e. composite of cells) (c).

In order to visualize the electroporation process as animations in three dimensions we used 3D Studio Max software. We visualized the introduction of small molecules through an electroporated cell membrane, into an electroporated cell and into all successfully electroporated cells within an exposed tissue (i.e. a composite of cells), as shown in Fig. 5.

**Figure 6:**
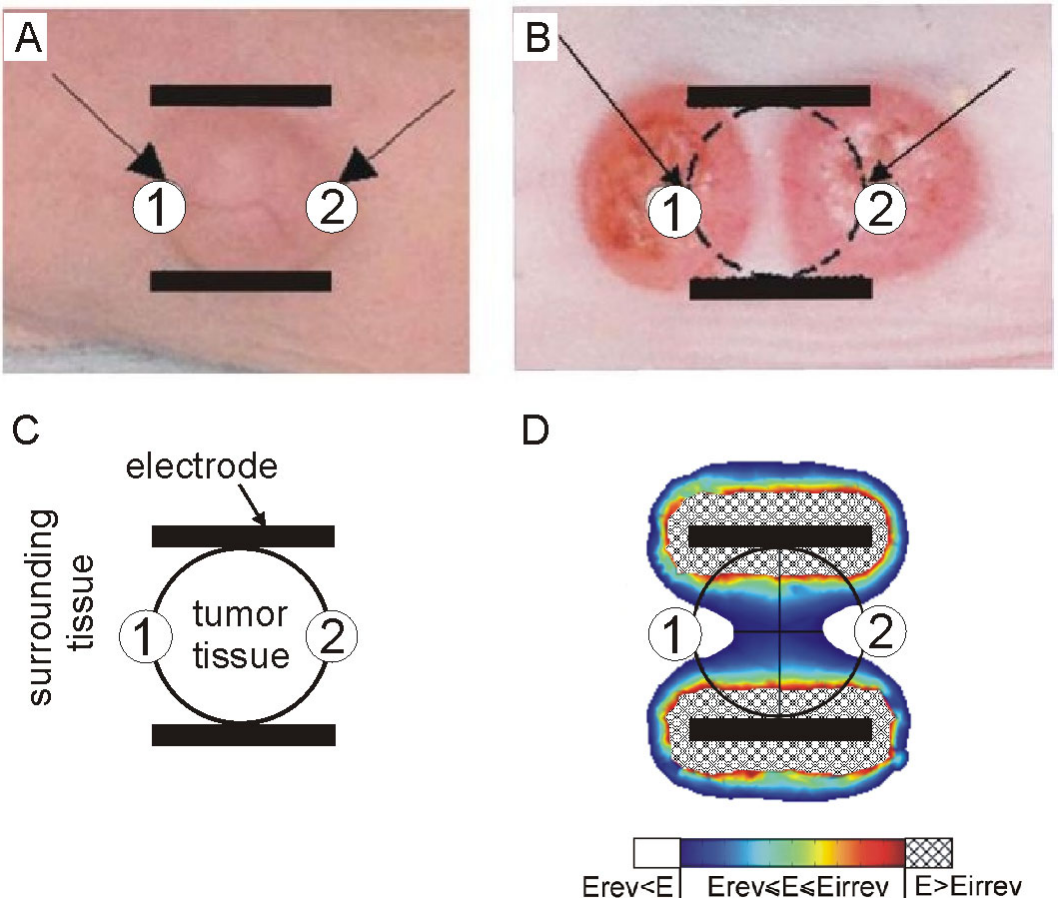
**In vivo electrochemotherapy performed on a nude mouse -- tumour regrowth after initial disappearance**. In vivo electrochemotherapy of tumour performed on a nude mouse: a) The electrode position and the tumour geometry just before the treatment, b) after the treatment two new tumours regrew in the regions (marked with numbers 1 and 2) where the tumour tissue was not exposed to *E *> *Erev*, c) graphical illustration of the tumour and its surrounding tissue position between electrodes and d) calculated local electric field distribution: the reversibly electroporated tissue is marked with colours (from blue to red), the tissue exposed to *E *> *Erev *is marked white and the patterned region represents the irreversibly electroporated tissue *E *> *Eirrev*.

The second part of the e-learning content (Modelling and visualization of local electric field) provides educational content about the importance of modelling and visualization of local electric field in electroporation-based treatments. The user is warned about possible errors that can be made while performing cell or tissue electroporation, such as insufficient amplitude of electric pulses or inadequate electrode geometry or electrode positioning. This part of e-learning content is particularly intended as guidance to the practitioners who perform electrochemotherapy treatment of solid tumours. Namely, for successful tumour treatment all the tumor cells have to be destroyed, otherwise the tumour cell can re-grow due to the insufficient magnitude of local electric field *E *<*Erev*. This was demonstrated in our e-learning application with an example of an unsuccessful subcutaneous tumour treatment performed on a nude mouse shown in Fig. 6. The Fig. 6a shows the electrode position and the tumour geometry just before the treatment, while Fig. 6b shows the regrowth of two tumours after initial disappearance: two new tumours regrew in the regions (marked with numbers 1 and 2) where the tumour tissue was not exposed to the sufficient electric field *E *>*Erev*. Simple graphical illustration of the tumor and its surrounding tissue position between two plate electrodes is shown in Fig. 6c. In Fig. 6d calculated local electric field distribution: the reversibly electroporated tissue is marked with colours (from blue to red), the tissue exposed to *E *>*Erev *is marked white and the patterned region represents the irreversibly electroporated tissue *E *>*Eirrev*.

By using simple graphical illustration we pointed out that the effectiveness of electrochemotherapy can be improved by: optimizing the applied voltage, changing electrode dimension or changing electrode orientation and their position, which we previously predicted by means of numerical modelling. We further provide a list of important parameters of the local electric field in electroporation-based treatments, such as: electrode geometry (needle or plate electrodes), dimension of the particular electrode (width, length, diameter), distance between electrodes, electrode position with respect to the target tissue, electrode orientation with respect to the target tissue, geometry of the target tissue, geometry of the tissue surrounding the target tissue, the contact surface between the electrode and the tissue, electric properties of the target tissue i.e. tissue conductivity, electric properties of the surrounding tissue, the voltage applied to the electrodes and threshold values of the tissue *Erev *and *Eirrev*. Using mathematical modelling and graphical illustrations we showed that the local electric field within the treated tissue is not homogeneous due to the specific structure and electric properties of the tissues (particularly of the target tumour tissue that usually has higher electric conductivity than its surrounding tissues).

In the third part of the e-learning application (Local electric field in 3D tissue models) we developed an interactive module for visualization of local electric field distribution in tissues for different parameters such as voltage applied, distance between electrodes, electrode' dimension and shape, tissue geometry and electric conductivity. The module provides 3D animations we developed by using 3D Studio Max, which were based on previously calculated local electric field distribution in 3D realistic tissue models. For the numerical calculations we used COMSOL Multiphysics software.

The module allows for local electric field visualization in cutaneous (protruding tumours) and subcutaneous tumours (tumours more deeply seeded in the tissue). Users can appreciate the local electric field distribution within the treated tissue when electroporated directly or through the skin by using plate or needle electrodes. The module also provides a guideline on how to overcome a highly resistive skin tissue in order to permeabilize more conductive underlying tissues.

**Figure 7:**
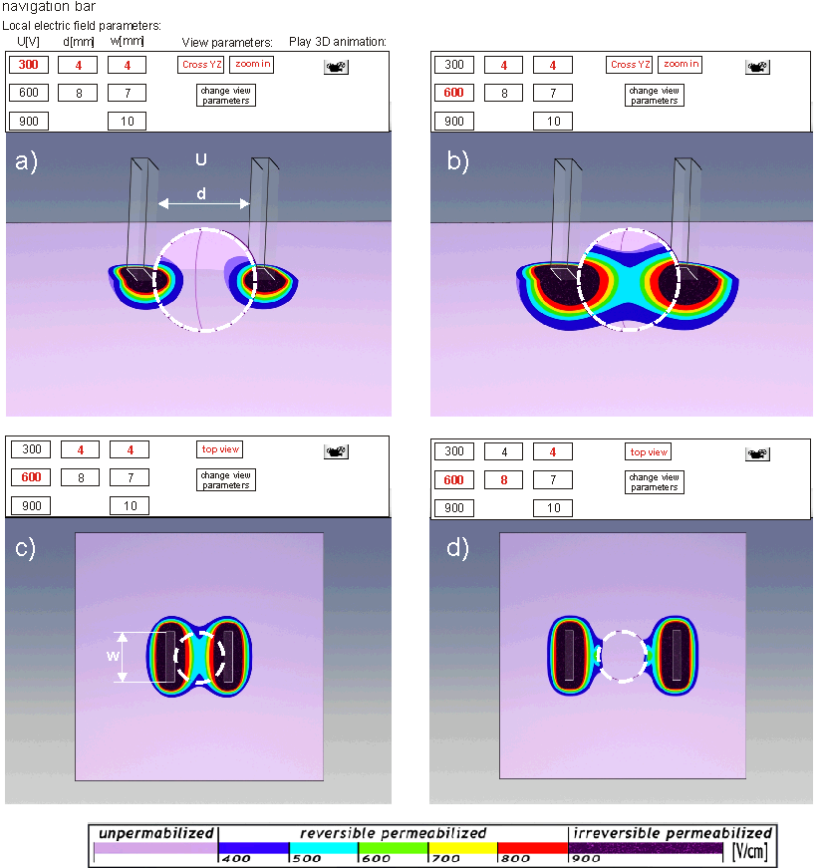
**Local electric field distribution within a model of cutaneous (protruding) tumour**. Local electric field distribution inside the protruding tumour model for two different applied voltages on the electrodes: a) *U *= 300 V and b) *U *= 600 V. The electrodes are 4 mm wide and 4 mm apart in both cases. Electric field distribution inside the models for two distances between electrodes: c) *d *= 4 mm and d) *d *= 8 mm. The electrodes are 4 mm wide with the applied voltage *U *= 600 V in both cases.

The objective of this part of the e-learning application is to provide an interaction with the educational content in order to simulate the "hands-on" learning approach about the parameters of the local electric field. By varying different parameters (such as amplitude of electric pulses, electrodes' dimensions and shape and distance between electrodes) in the navigation bar users have the possibility to shape the electric field distribution within the models (see the navigation bar in Fig. 7). The local electric field distribution can be viewed in 2D model cross-sections or played as a 3D animation. The *E *is displayed in the range between *Erev *to *Eirrev*. In Figure 7 the local electric field distribution inside the cutaneous protruding tumour obtained with two different amplitudes of applied voltage (Fig. 7a: *U *= 300 V and Fig. 7b: *U *= 600 V) using two parallel plate electrodes is shown as example. By increasing the applied voltage (for the same tissue geometry, electrode size and position) the stronger local electric field is obtained. Similar effect can be achieved by increasing the electrode dimensions (electrode width), while by increasing the distance between electrodes the tumor is exposed to a lower local electric field intensity, as shown in Figs. 7c and 7d.

**Figure 8:**
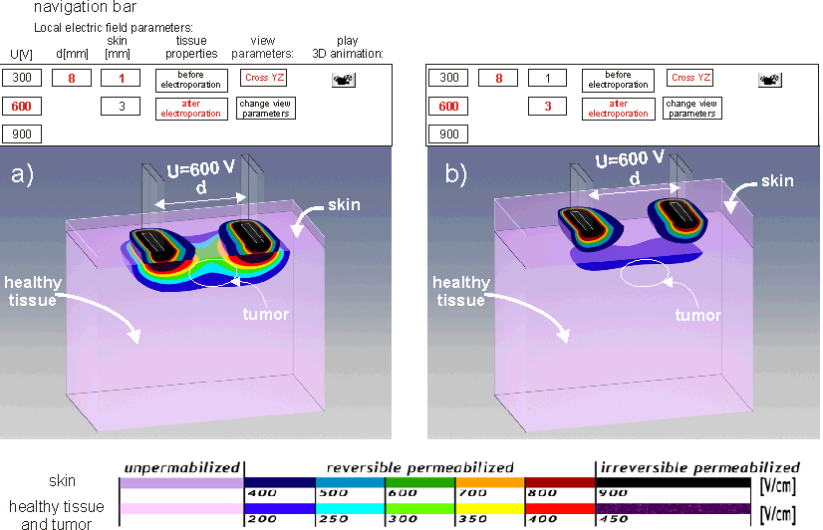
**Local electric field distribution within a model of subcutaneous tumor**. Local electric field distribution within a model of subcutaneous tumour seeded below: 1 mm thick skin layer (a) and 3 mm thick skin layer (b).

The model of subcutaneous tumour gives the user an insight into the local electric field within the target tissue when electroporated through the skin. This model is composed of two layers; the upper layer representing skin tissue with lower specific conductivity compared to the underlying layer which is more conductive. The electric field distribution is presented in two models with two different thicknesses of the skin layer: 1 mm (Fig. 8a) and 3 mm (Fig. 8b). Thus, the user can appreciate the presence of the skin and its poor electric conductivity on the local electric field distribution within the target tumour and its surroundings.

**Figure 9:**
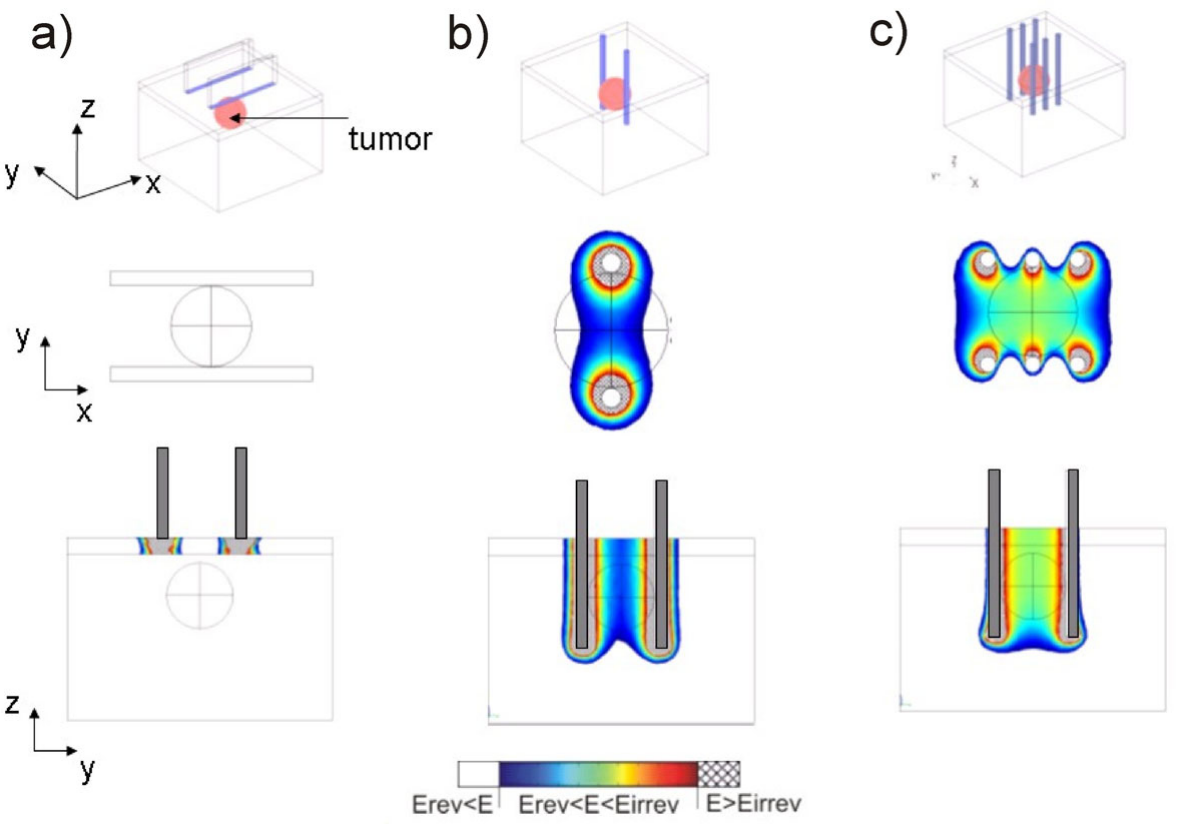
**Local electric field in subcutaneous tumour model obtained by using different electrode configurations**. Local electric field distribution in subcutaneous tumour model using plate electrodes (a), a pair of needles (b) and three pairs of needles (d). The applied voltage in all cases was *U *= 300 V.

The key messages that the interactive module provides are:

1) in order to successfully electroporate the target tumour through the skin layer a higher voltage needs to be applied compared to the tumour electroporation, which further depends also on skin thickness. The user is offered a guideline on how to overcome the highly resistive skin tissue in order to permeabilize more conductive underlying tissues using plate electrodes (Fig. 8);

2) plate electrodes are more suitable for treatment of protruding cutaneous tumours, while for situations when the tumour is seeded more deeply in the tissue needle electrodes are to be used (Fig. 9a and 9b), and;

3) by increasing the number of needle electrodes stronger local electric field in the tissue can be achieved (Fig. 9c).

The educational web pages are concluded by a test (see Additional file 1) that gives the user an opportunity to test the acquired knowledge, while allowing the teacher and the web-developer to follow the efficacy of the constructed pages and their educational success.

### Results of the pedagogical efficiency evaluation

**Figure 10:**
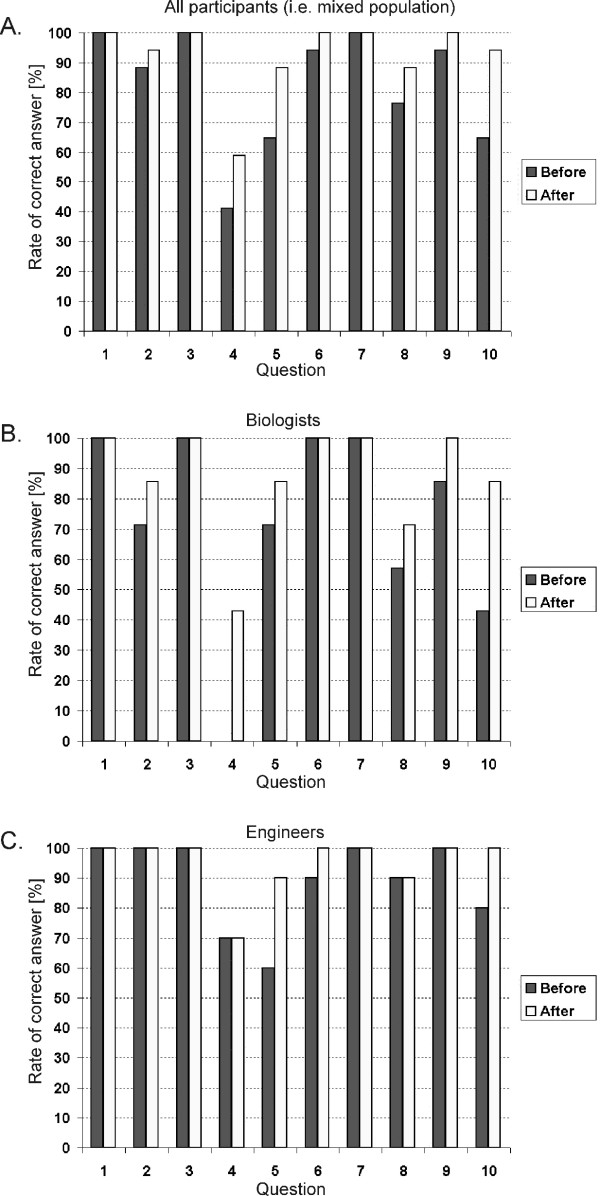
**Results of the pedagogical efficiency evaluation**. Percentage rate of correct answers for each question analyzed for: a) all participants (i.e. mixed population); b) biologists and c) engineers. The questions' numbering corresponds to the question' numbering in the test given in Additional file 1.

The results of the pedagogical efficiency evaluation are shown in Fig. 10. The evolution of the scores obtained from the test before and after the e-learning session was analyzed on the basis of each question (listed in Additional file 1), which allowed for testing the participants' knowledge improvement for each question and the pertinence of the questions. The results of percentage rate analysis of correct answers to each question of the pre and post e-learning session test given by all participants, (both participant groups i.e. engineers and biologists), is shown in Fig. 10a. The results of the percentage rate analysis done for biologists and engineers separately are shown in Figs. 10b and 10c, respectively.

The percentage rate of correct answers for all participants (mixed population) obtained after the e-learning session was above 50% for all questions in the test (Fig. 10a). The results in Fig. 10a show that the level of knowledge of all participants was improved after the e-learning session compared to the knowledge shown before the session. The results in Fig. 10b show that before the e-learning session the knowledge of biologists was more heterogeneous compared to the knowledge possessed by engineers as shown in Fig. 10c.:

1) for biologists the average percentage rates of correct answers changed from 73% before e-learning session to 87% after e-learning session, with a large dispersion depending on the question (for example - question 4: from 0% before to 43% after e-learning session; question 9 from 86% before to 100% after e-learning session), Fig. 10b.

2) for engineers the average percentage rates of correct answers changed from 89% before e-learning session to 95% after e-learning session, Fig. 10c.

Nevertheless, increase in percentage rate of correct answers, after the e-learning session, to each of the questions was obtained for both groups i.e. biologists and engineers (Figs. 10b and 10c).

### Results of the usability evaluation

**Figure 11:**
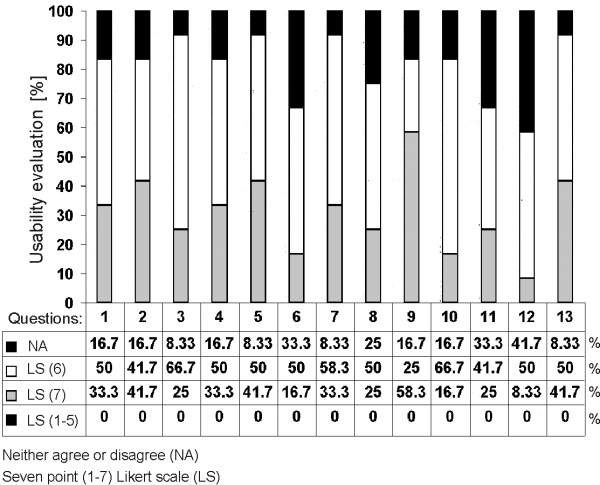
**Results of the usability evaluation**.

The results of the usability evaluation of the e-learning application are shown in Fig. 11. The participants expressed their opinion for all 13 usability related questions with 6 or 7 agree statements in the seven point Likert scale (LS (6) and LS (7)) and with neutral neither agree nor disagree statement (NA). None of the questions was evaluated with statements from 1 to 5 in the Likert scale (LS (1-5)), as shown in Fig. 11.

The participants evaluated the statement that the information provided by the system is easy to understand (question 9) with the highest percentage of agree statements in the Likert scale (58.3% of LS (7) and 25% of LS (6)). Participant were most neutral (41.7% of NA) regarding question 12 (The system covers all the areas I expected to cover). However, the same question was evaluated with 50% of LS (6) and 8.3% of LS (7) statements. The participants were neutral with 33% for questions 6 (I believe I became more confident with the system) (with 50% of LS (6)) and for question 11 (The interface of system is pleasant) (with 41.67% of LS (6)). Overall, the participants were satisfied with the developed e-learning application (question 13) with 41.6% of the highest percentage of agree statements in the Likert scale (LS (7)) and with only 8.3% of neutral statements (NA). The results of the usability evaluation (Fig. 11) also revealed that the participants were satisfied with how easy it was to use the system (question 1: 33.3% of LS (7) and 50% of LS (6)). The participants were comfortable using the system (question 4: 33.33% of LS (7) and 50% of LS (6)) and found the system simple to use (question 2: 41. 67% of both LS (7) and LS (6)), to learn to use (question 5: 41.67% of LS (7) and 50% of LS(6)) and to be effectively navigated (question 3: 25% of LS (7) and 66.67% of LS (6)). The users also found the information provided with system (such as online help, on-screen messages, and other documentation) clear (question 7: 33.3% of LS (7) and 50% of LS (6)), easy to find (question 8: 25% of LS (7) and 50% of LS (6)) and effective and complete (question 10: 16.67% of LS (7) and 66.67% of LS (6)) (Fig. 11).

After completing the usability questionnaire the participants provided their opinion/comments on the application and suggestions on its improvement. Most of participants provided the comment that they liked the idea to present the knowledge on electrochemotherapy in the form of e-learning application. The participants particularly found interesting the interactive visualization of local electric field in tissues for different parameters such as voltage applied, distance between electrodes, electrode' dimension, which for the time being can not be visualized while performing the electrochemotherapy treatment. The engineers, who are not familiar with chemical and biological processes during electroporation of cells and tissues, suggested that more of biological and chemical background should be also added to the existing educational material. On the other hand the biologists suggested that it would be interesting to have a possibility to visualize the distribution of local electric field and changes in electric properties for different cell types such as muscle fibers, hepatocytes, blood vessels, while being electroporated and which are potential target cells for gene transfer.

## Discussion

We developed, implemented and evaluated an e-learning application on electroporation-based therapies such as electrochemotherapy. This is the first e-learning application developed to support collaboration, knowledge and experience exchange among the experts from different scientific fields involved in electrochemotherapy and other electroporation-based therapies and in order to organize and to transfer the acquired knowledge and experience to the users (such as clinicians, medical personnel, students, patients and all those who want to learn about electroporation-based therapies).

The educational content on electrochemotherapy and cell and tissue electroporation is based on previously published studies from molecular dynamics, lipid bilayers, single cell level and simplified tissue models to complex biological tissues and research and clinical results of electrochemotherapy treatment [3,4,11,31-43].

The e-learning content presents three different levels of knowledge on cell and tissue electroporation. In the first part of the e-learning application we explain basic mechanisms underlying electroporation process. Based on simple graphical illustrations we demonstrated the influence of each of the pulse parameters, such as pulse amplitude, pulse number and duration, on electroporation of cells with different sizes, shapes and orientations with respect to the applied electric field. By using 3D animation we visualized the aqueous pore formation in cell membrane, which is most widely accepted model, among different theoretical models that describe cell membrane electroporation.

Electrochemotherapy treatment outcome is directly related to the local electric field distribution within the target tumour tissue and its surrounding tissues [11,12,40,47-49]. The second part of the e-learning content was thus developed in order to provide the educational material about the parameters of local electric field being crucial to make the tumor treatment as efficient as possible. For this purpose we used combination of numerical calculations by means of mathematical modelling and simple graphical illustrations. We demonstrated how the pulse amplitude, electrode shape and electrode positioning influence on the local electric field distribution within the treated cells and tissues. We also demonstrated how the electric properties of a treated sample (i.e. its geometry and electric conductivity) can modify the local electric field distribution. Namely, when the voltage is applied, the electric field distributes within the complex tissue with different electric properties as in voltage divider. The latter means that the electric field is the highest in the layer with the highest electric resistivity (lowest conductivity) [43], which is particularly important when electroporating the skin and/or its underlying tissues.

In the third part of the e-learning application, we developed an interactive module for visualization of local electric field distribution in tissues for different parameters such as voltage applied, distance between electrodes, electrode' dimension, tissue geometry and electric conductivity. The interactive module is aimed at hands-on learning on how the above-mentioned parameters can modify the local electric field distribution within the treated tissue. The module allows for local electric field visualization in cutaneous and subcutaneous tumours. Users can appreciate the local electric field distribution within the treated tissue when electroporated directly or through the skin by using plate or needle electrodes. The module also provides guidelines on how to overcome a highly resistive skin tissue in order to permeabilize more conductive underlying tissues. Since, for the time being the local electric field in the treated tissue can not be visualized while performing the electrochemotherapy treatment, the interactive visualization approach we provide in our e-learning application can serve as an important tool in selection of the appropriate electric pulses amplitude, electrode shape and their placement with respect to the tissue geometry and its electric conductivity, which is needed for best electrochemothertapy treatment outcome.

Good collaboration among the participants and with the instructor was established during the e-learning session. Namely, the participants assisted each other while studying the educational content and several discussions were initiated between physicists and biologists and between the participants and the instructor. The e-learning application was concluded by a test on the presented educational material and by a questionnaire on usability of the developed application.

We evaluated the designed e-learning application at the International Scientific workshop and postgraduate course (Electroporation Based Technologies and Treatments) [45]. The evaluation was carried out by testing the pedagogical efficiency of the presented educational content and by performing the usability study of the application. The pedagogical efficiency assessment showed that the participants improved their level of knowledge (Fig. 10).

The percentage rate of correct answers for all participants (mixed population) obtained after the e-learning session was above 50% for all test questions (Fig. 10a). The results in Fig. 10b show that before the e-learning session the knowledge of biologists was more heterogeneous compared to the knowledge possessed by engineers as shown in Fig. 10c. This is in part because the level of knowledge possessed by biologists (compared to the engineers) was lower before the e-learning session, since the test and the e-learning content was about electrical parameters. However, the increase in percentage rate of correct answers, after the e-learning session compared to the results obtained before the e-learning session, to each of the questions was obtained for both biologists and engineers (Figs. 10b and 10c). Only for question 4 the percentage of correct answers given by biologist after the e-learning session was slightly below 50% (i.e. 43% of success rate after e-learning session compared to 0% before e-learning session). In order to further improve the success rate of question 4 we concluded that: 1. question 4 should be more clearly formulated by developers and 2. more of e-learning content on the voltage applied between electrodes (*U*) and on electroporation threshold of local electric field (*E*) should be provided in the e-learning application. The same question answered by engineers was 70% of success rate before and after e-learning session.

The results of usability evaluation revealed that participants found the application simple to learn to use and navigate (Fig. 11). Overall, the participants were satisfied with the e-learning application. The participants found the information provided by system easy to understand (question 9 with the highest percentage of agree statements in the Likert scale (58.3% of LS (7)) and 25% of LS (6)). The participants were most neutral regarding the statement that the e-learning application covered all the areas they expected to cover (question 12 evaluated with 41.7% of NA). However, the same question was evaluated with 50% of LS (6) and 8.3% of LS (7) statements. The modular structure of the application allows for upgrade with new educational content collected from the clinics and research, and for the integration of new application modules including computer-supported collaborative visualization being an important component in remote collaboration among the experts [29]. The e-learning application can be used as an education form at both levels: either as a completely independent e-learning form or as an integral part of a blended learning form. The e-learning session can be executed by the users in a linear sequence of studying steps according to the program flow model (i.e. by starting at the beginning of the e-learning content and by concluding with the final evaluation tests) or in a studying sequence which is not previously defined, which can serve as an additional e-learning module of blended learning [30].

## Conclusion

The e-learning application together with E-CHO system is available to the users to provide collaborative and flexible learning in order to facilitate knowledge exchange among the experts from different scientific fields that are involved in electrochemotherapy. The e-learning application is developed to provide an interactive educational content in order to simulate the "hands-on" learning approach about the parameters being important for successful therapy. The e-learning application on electrochemotherapy can be easily adapted to serve as a collaborative e-learning tool also in other electroporation-based treatments such as gene electrotransfer, irreversible tissue ablation or transdermal gene and drug delivery [6,8,9,50]. The presented e-learning application provides an easy and rapid approach for information, knowledge and experience exchange among the experts from different scientific fields, which can facilitate development and optimisation of electroporation-based treatments.

## Competing interests

The authors declare that they have no competing interests.

## Authors' contributions

All authors read and approved the final manuscript.

## Supplementary Material

Additional file 1 **Pedagogical efficiency questionnaire**. The file provides the test on the educational content completed by the participants before and at the end of the e-learning session. Click here for file

Additional file 2 **Usability efficiency questionnaire**. The file provides usability questions related to the user satisfaction with the developed e-learning application. Click here for file
