# Supplementary material for: An e-learning application on electrochemotherapy
Source: Biomed Eng Online. 2009 Oct 20;8:26. doi: 10.1186/1475-925X-8-26 (PMC2770511; doi:10.1186/1475-925X-8-26)
Supplement: Additional file 1 — Pedagogical efficiency questionnaire. The file provides the test on the educational content completed by the participants before and at the end of the e-learning session. [file 1475-925X-8-26-S1.PDF]

APPENDIX 1

QUESTIONS

1. The requirement for effective electrochemotherapy in terms of local electric field distribution E is the following:

a) entire tumor tissue has to be exposed to E above reversible threshold Erev
b) entire tumor tissue has to be exposed to E below reversible threshold Erev

2. Which parameters have to be optimized in order to obtain the appropriate local electric field inside the tumor and its surrounding healthy tissues?

a) electrode geometry
b) position of electrodes
c) applied voltage
d) the dose of bleomycin

(Attention: multiple right answers are possible)

3. A spherical cutaneous tumor (2r = 4 mm) shown in Figure 1 has to be treated with ECT using 2 parallel plate electrodes. Currently applied voltage (U=300 V), distance between electrodes (8 mm) and electrode dimensions (4 mm wide) are not appropriate parameters. In order to obtain higher local electric field distribution inside the tumor the distance (d) between electrodes has to be:

a) decreased
b) increased

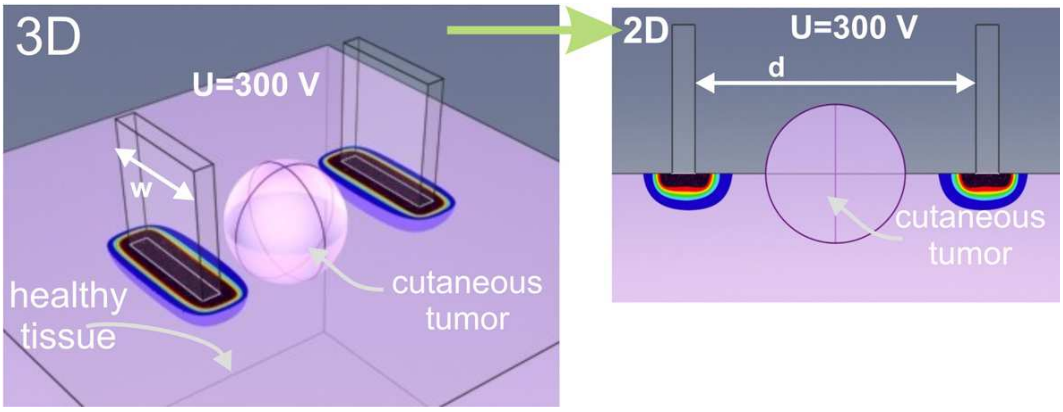

Figure 1: Electric field distribution within 3D and 2D models of cutaneous tumor with plate electrodes, where distance between electrodes d = 8 mm, electrode width w = 4 mm and the applied voltage U = 300 V

4. A pair of plate electrodes you are going to use are 10 mm wide (w = 10 mm). If the distance between electrodes is 6 mm, the largest area of the tumor shown in Figure 1 will be exposed to the electric field above Erev = 400 V/cm, when you apply

a) U= 300 V
b) U= 600 V
c) U= 900 V

5. You can choose among 4, 7 or 10 mm wide pairs of electrodes. When the distance (d) between the electrodes and the applied voltage (U) are fixed at d = 5 mm and U = 900 V the entire tumor tissue (see Figure 1) is exposed to E ≥ Erev. Which pair of electrodes would you use in order to prevent damages to the healthy tissue?

a) w= 4 mm
b) w= 7 mm
c) w= 10 mm

6. If the plate electrodes are too short with respect to the tumor tissue (see Figure 2) or the applied voltage on the electrodes is too low the following tumor response can be expected:

a) tumor cells regrow in the central region of the tumor (Region 1)
b) tumor cells regrow in close proximity of the electrodes (Region 2)
c) tumor cells regrow in regions which are far from the electrodes (Region 3)

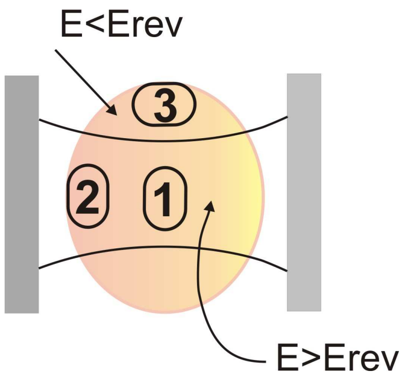

Figure 2: Illustration of a tumor with Regions 1 and 2 subjected to the E above Erev and Region 3 subjected to E below Erev

7. In order to obtain the same value of local electric field inside both subcutaneous tumors shown in Figures 3a and 3b, the applied voltage in the second case (Figure 3b) has to be:

a) U= 300 V
b) U= 900 V

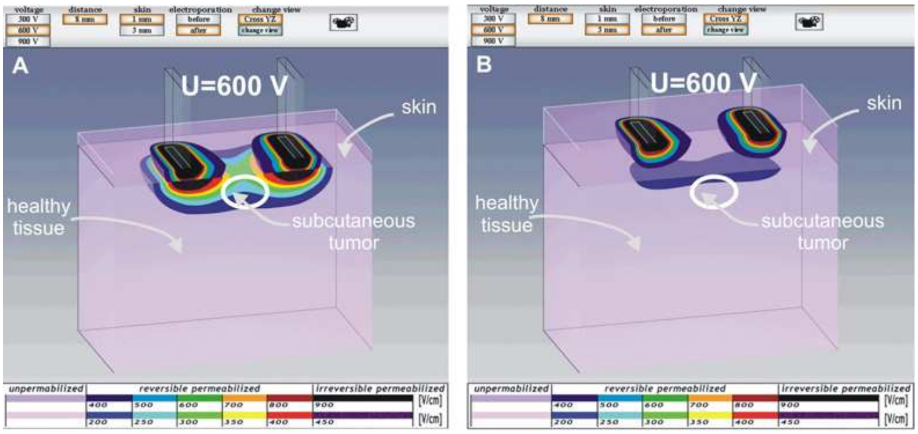

Figure 3: Electric field distribution within a model of subcutaneous tumors seeded: A. below a 1 mm thick skin layer; B. below a 3 mm thick skin layer

8. Due to electropermeabilisation of the skin layer, local electric field within underlying tissues:

a) increases
b) decreases

9. Which of the following needle electrode configurations will result in higher local electric field inside the subcutaneous tumors shown in Figures 3 (if the applied voltage U is the same in both cases)?

a) two needle electrodes (one-pair)
b) a parallel array of six needle electrodes (three-pairs)

10. See the electric field distribution within the target subcutaneous tumor seeded below a 3 mm thick skin layer in Figure 3b. If you had only the following two possibilities to increase the local electric field inside the tumor:

a) increase the applied voltage to U= 900 V
b) insert a parallel array of six needle electrodes (three-pairs) and apply U = 300 V

Which one would you choose?

I allow that the results of the electrochemotherapy e-learning test can be used for research purposes. All personal data will be kept anonymous.
