# Supplementary material for: An e-learning application on electrochemotherapy
Source: Biomed Eng Online. 2009 Oct 20;8:26. doi: 10.1186/1475-925X-8-26 (PMC2770511; doi:10.1186/1475-925X-8-26)
Supplement: Additional file 2 — Usability efficiency questionnaire. The file provides usability questions related to the user satisfaction with the developed e-learning application. [file 1475-925X-8-26-S2.PDF]

Appendix 2: Usability evaluation questionnaire

<u>Electrochemotherapy training Usability Questionnaire</u>

System: Electrochemotherapy training (e-learning application)

			1	2	3	4	5	6	7		NA
1.	Overall, I am satisfied with how easy it is to use **system**	**DISAGREE**								**AGREE**	
2.	It was simple to use **system**	**DISAGREE**								**AGREE**	
3.	I can effectively navigate the **system**	**DISAGREE**								**AGREE**	
4.	I feel comfortable using **system**	**DISAGREE**								**AGREE**	
5.	It was easy to learn to use **system**	**DISAGREE**								**AGREE**	
6.	I believe I became more confident using **system**	**DISAGREE**								**AGREE**	
7.	The information (such as online help, on-screen messages, and other documentation) provided with **system** is clear	**DISAGREE**								**AGREE**	
8.	It is easy to find the information I needed	**DISAGREE**								**AGREE**	
9.	The information provided for **system** is easy to understand	**DISAGREE**								**AGREE**	
10.	The information is effective and complete	**DISAGREE**								**AGREE**	
11.	The interface of **system** is pleasant	**DISAGREE**								**AGREE**	
12.	**system** covers all the areas I expect to cover	**DISAGREE**								**AGREE**	
13.	Overall, I am satisfied with **system**	**DISAGREE**								**AGREE**	
			1	2	3	4	5	6	7		NA
